# Influence of Process Aids on Solid–Liquid Interfacial Properties of Three-Component Hydroxyl-Terminated Polybutadiene Propellants

**DOI:** 10.3390/polym17030286

**Published:** 2025-01-23

**Authors:** Xulong Zhang, Zitong Deng, Wenlong Xu, Liping Jiang, Huixiang Xu, Qiufan Tang, Qilong Zheng, Jizhen Li

**Affiliations:** 1 Xi’an Modern Chemistry Research Institute, Xi’an 710000, China; xlzhang0116@163.com (X.Z.); d_zitong@163.com (Z.D.); xuwenlong204@163.com (W.X.); jiangliping204@163.com (L.J.); xhx204@163.com (H.X.); qiufantang@126.com (Q.T.); zhengqilong2577@163.com (Q.Z.); 2 School of Material Science & Engineering, Beijing Institute of Technology, Beijing 100000, China

**Keywords:** rheology, HTPB propellants, process aids, interfacial chemistry, polyene polyamines

## Abstract

The effect of the process aid “OPS” on the rheological properties of hydroxyl-terminated polybutadiene propellant was investigated by formulating different components of high-solid-content slurry, and the change in slurry viscosity with shear rate, surface morphology of solid-phase particles, and contact angle of the relevant interfaces were characterized. The results showed that the polyalkene polyamine surfactant OPS could significantly reduce the apparent viscosity and enhance the rheological properties of the slurry, to up to a 30% reduction, and the effect was achieved by adjusting the interfacial properties of the aluminum powder and the binder system. With the addition of 0.1% OPS, the contact angle of the interface between the aluminum powder and the binder was obviously reduced, from 97° to 30°, and the wetting was significantly enhanced, so it was judged that the OPS was suitable for HTPB-based composite propellants.

## 1. Introduction

Composite solid propellants are a class of solid propellants consisting of binder systems (including adhesives, plasticizers, curing agents, etc.), oxidizers, metal fuels, nitramine explosives, and process aids. According to the different types of binders, they can generally be divided into polysulfide rubber (PS) propellant, polyvinyl chloride (PVC) propellant, polyurethane (PU) propellant, and various types of polybutadiene (PB) propellant. PB propellants, especially hydroxyl-terminated polybutadiene (HTPB) propellants, i.e., butyl hydroxyl propellants, have significantly better properties and are the focus of the current development of composite solid propellants [1,2,3].

In recent years, the development and application of HTPB propellant has been advanced [4]. Energy performance is one of the most important indicators of propellant effectiveness. For HTPB propellants, the solid component content is highly positively correlated with energy performance. However, with the increase in solid component content, the friction between solid particles increases dramatically, and the rheological properties of the slurry decrease significantly, which leads to the failure of the propellant process. Limited by the rheological properties, current composite propellants often do not contain more than 88% solid components. Therefore, rheological properties are an important factor limiting the energetic performance of propellants [5].

Composite solid propellants have a wide variety of components, and the factors affecting the rheological behavior of their slurries are also more complex. In the three-component HTPB propellants, the mass fraction of solid-phase particle component ammonium perchlorate (AP) and aluminum powder (Al) is very high, and the solid content often reaches more than 85%. AP is an important oxidizer in composite propellants that supplies oxygen during the combustion process. And Al powder as a metal fuel contributes greatly to the energy performance of the propellant. HTPB acts as a polymer binder to bond the other components and provides the necessary mechanical properties for the propellant after curing. The high content of HTPB, AP, and Al forms a complex and diverse interface, in which the phase interface between the particles and the liquid phase is an important factor affecting the rheological properties of the slurry [6,7]. There have been many studies on the use of process aids to improve the rheological properties of fluids [8,9]. Process aids are an important part of composite solid propellant formulations, which are the key functional components used to regulate the rheological properties of propellant slurries and improve process performance, and mainly play the roles of lowering the viscosity of the slurry, reducing the yield stress, improving the flow and leveling properties of the slurry, and prolonging the slurry applicability period [10]. Han [11,12] introduced the surfactant SU-2 into an HTPB system, and the initial viscosity of the blank formulation in the experiment was 607 Pa·s, while the initial viscosity of the formulation containing 0.05% SU-2 was 396 Pa·s. Wang [13] tested 14 common surface-active substances in the ADN system, and the results showed that most of the surface-active substances could reduce the viscosity of the slurry, among which the effect of lauryl amine was the most significant, which could reduce the viscosity of the slurry to 40% of the blank formulation. Tang [14] found that capping the binder with urethane was beneficial in improving the wettability of the filler surface, and the viscosity of the urethane-modified control group decreased by 62% compared to the blank formulation. Wen [15] investigated the effect of hyperbranched polyester MHBPE on the rheological properties of slurry and found that the addition of this type of additive could effectively reduce the viscosity of slurry by up to 40% at all temperatures. It was hypothesized that under the action of this additive, the hyperbranched polymer chains formed by the cross-linking of macromolecules had large free space for particles to flow freely, thus reducing the viscosity of the slurry.

Current research on process aids mainly focuses on the synthesis and evaluation of their effects, and there is a relative lack of research work on solid-phase particles in slurries from the perspective of phase interface [16,17,18]. In this study, the surfactant OPS (Optimized Polyene polyamine Surfactant) was selected to determine the mechanism of action of process aids by testing the interfacial parameters between various aids and the solid-phase particles in the three-component HTPB propellant, and to propose the selection of process aids for the three-component HTPB propellant for the improvement of rheological properties in slurries.

## 2. Experiment

### 2.1. Experimental Materials

We acquired the following: AP, class III (particle size 100–140 mesh), with fine AP (particle size 6–8 μm) included, provided by Dalian Gaojia Chemical Co., Dalian, Liaoning province, China; Al, 29 μm, provided by ANSTEEL Group, Anshan, Liaoning Province, China; HTPB, hydroxyl value 0.68 mmol/g, number average relative molecular mass 3100, respectively, provided by Liming Chemical Research and Design Institute Co., Luoyang, Henan province, China; Diisooctyl sebacate (DOS), chemically pure, provided by Yonghua Chemical Co., Changshu, Jiangsu province, China; and surfactant OPS, provided by Xi’an Modern Chemistry Research Institute, Xi’an, Shaanxi province, China.

### 2.2. Preparation of Slurries

The binder HTPB and plasticizer DOS were mixed uniformly at a ratio of 3:1 to make a blank liquid system for the experimental slurry; the mass ratio of solid-phase particles required for the experiment was calculated, according to the closest-packing model, to be class III AP/Al/fine AP = 74:26.2:9.3 [19], and the solid content of the slurry was 84%; the solid-phase particles were placed in a 50 °C oven for drying and were added into a blank liquid system in order of their average particle sizes, from large to small, and each particle was stirred at a constant temperature of 40 °C until the slurry was uniformly dispersed, which was labeled as a blank formulation of the slurry; the solid-phase particles were processed unchanged, and the control slurry was made by adding a 0.1% mass fraction of OPS solution in the liquid formulation stage, respectively.

Class III AP and Al were selected to research the interfacial properties of different solid-phase particles and different liquid systems, and the abbreviations and components of the slurries prepared are shown in Table 1, in which the mass ratio of HTPB, DOS, and solid-phase particles is 3:1:5 for the slurries labeled “Al-containing liquid” and “AP-containing liquid”, and the mass ratio of HTPB and DOS is 3:1 for the slurries labeled “liquid”. In the slurry labeled “Al-containing liquid” and “AP-containing liquid”, the mass ratio of HTPB, DOS, and solid-phase particles was 3:1:5, and in the slurry labeled “liquid”, the mass ratio of HTPB and DOS was 3:1; and, if the process aids were included, the mass percentage of process aids was 0.1%.

### 2.3. Experimental Apparatus and Interfacial Property Testing of Slurries

The viscosity–shear rate curves of all the slurries in Table 1 were tested using a HAAKE MARS rheometer, Thermo Fisher Scientific, Waltham, MA, USA, at a constant temperature of 50 °C, which is a temperature that is close to the actual working conditions, with a shear rate range of 0–20 s^−1^, using linear point-taking, CR mode, and flat-plate fixture, with the fluid lying flat in the fixture during the test, and a rotor made of PP35Ti. This was done at room temperature (25 °C) using a DSA30S contact angle tester, A.Kruess Optronic GmbH, Hamburg, Germany, for class III AP and an Al static contact angle test; static contact angle testing was performed when the test reference liquid directly in the form of droplets titrated on the spread of particles. The results are very intuitive; powder samples were spread on a slide and placed on the contact angle test bench, respectively, using the liquid group of a variety of slurries for contact angle test reference liquid with a probe to complete the droplet and powder sample (respectively, take class III AP and Al for testing) contact, the droplet contact samples, and using the droplet probe immediately after the photo was taken. The instrument software automatically calculates the contact angle of the sample and takes photos according to the noise reduction and other processes, allowing for observations of the contact angle.

## 3. Results and Discussion

### 3.1. Effect of Process Aids on the Rheology of Slurry

Viscosity describes the internal resistance of a fluid to flow. The viscosity of Newtonian fluids does not change with change in shear rate, while, for a propellant slurry, as a typical non-Newtonian fluid, its shear rate–viscosity curve reflects the apparent viscosity with the change rule of shear rate, which is an important basis for the determination of the rheological properties of the solid propellant slurry. It directly affects the process performance, and especially the casting processability [20]. Within a certain range, it is thought that the lower the viscosity of the slurry, the better the rheological properties [21].

**Figure 1:**
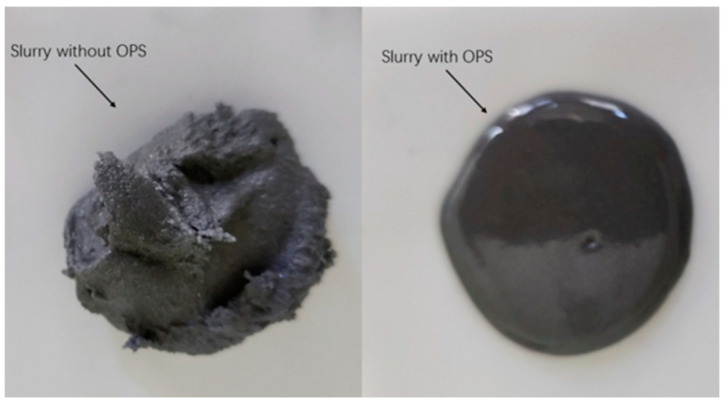
Macroscopic morphology contrast of slurry.

The macroscopic morphology of the two slurries (Figure 1) was observed after 24 h of resting. It was found that the full-component formulations could not be naturally leveled at room temperature (25 °C), while the full-component formulations/OPS slurries could be naturally leveled, and the surface of the slurries was smooth and basically free of air holes, which could indicate that they had good leveling properties. Combined with the rheology curve data below, it can be judged that the surfactant OPS can effectively improve the rheological properties of HTPB propellant.

The shear rate available during the actual processing of the propellant slurry is about 1–15 s^−1^. In order to ensure that the rheological curves present a complete characterization of the pseudoplastic fluid with high solid content, a shear rate range of 0–30 s^−1^ was selected for the rheological testing of the blank formulation slurry and the 0.1% OPS slurry, and the resulting viscosity–shear rate curves are shown in the following figure.

**Figure 2:**
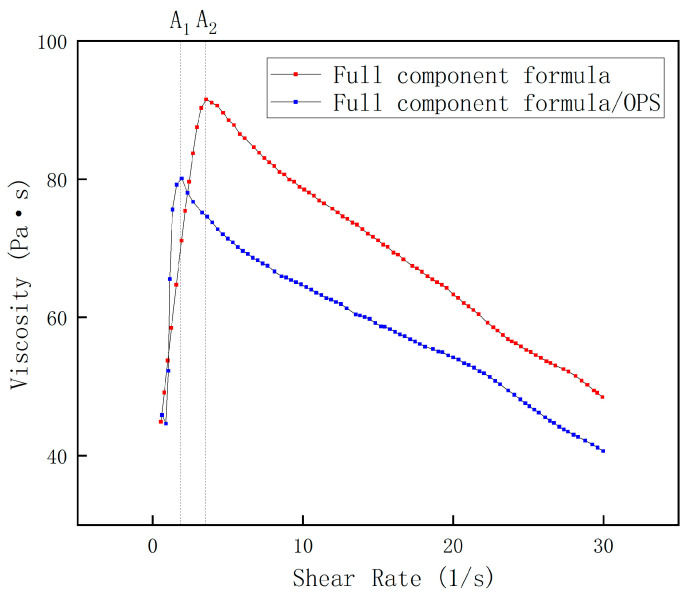
Slurry viscosity of full-component formula.

It can be seen that the two curves in Figure 2 show an obvious “rising and falling” trend, showing the common characteristics of polymer fluids. This is mainly because when the shear rate is small, in the graph line up to points A_1_ and A_2_ in the figure, the fluid in the macromolecular components of HTPB in the shear effect of entanglement results in a sharp increase in resistance to intermolecular movement, which occurs with the viscosity peak before the surge in viscosity. As the shear stress increases linearly with the shear rate, after the two graph lines reach A_1_ and A_2_, respectively, with the increase in shear rate, the macromolecular chain is subjected to shear force, such that the intermolecular entanglement is broken. The macromolecular chain segments begin to be arranged in a certain orientation along the direction of the shear stress, the resistance to chain segment movement is dramatically reduced, and the viscosity decreases with the increase in shear rate. As can be seen from the figure, the viscosity of the slurry with OPS is significantly lower than that of the slurry without OPS, both at the apex of the curve and after the apex. The maximum values of the viscosity curves of the slurries with and without OPS were 78.53 Pa·s and 99.79 Pa·s, respectively, and the average values of the viscosity curves were 63.83 Pa·s and 74.49 Pa·s, respectively, and it was obvious that the addition of OPS affected the viscosity of the slurries to a greater extent. It is worth noting that, after the shear rate exceeds 20 s^−1^, the viscosity may change irregularly with the change in shear rate, because under at 50 °C, the shear rate is too large to lead to the slipping between the fixture and the liquid surface of the liquid. Thus, the fixture idles, which ultimately results in the apparent viscosity of the test, but cannot truly reflect the rheological situation of the slurry.

It is generally believed that surfactants reduce yield value and viscosity by improving the wetting of the filler interface with the binder system, allowing the filler to be easily dispersed and breaking up the cohesive agglomerates that may be present in the original slurry [22]. A major key to studying the mechanism of surfactant action on propellant slurries is to determine which interface or interfaces in the slurry system the surfactant mainly acts on.

### 3.2. Mechanism of Surface-Active Substances to Improve Rheological Properties

**Figure 3:**
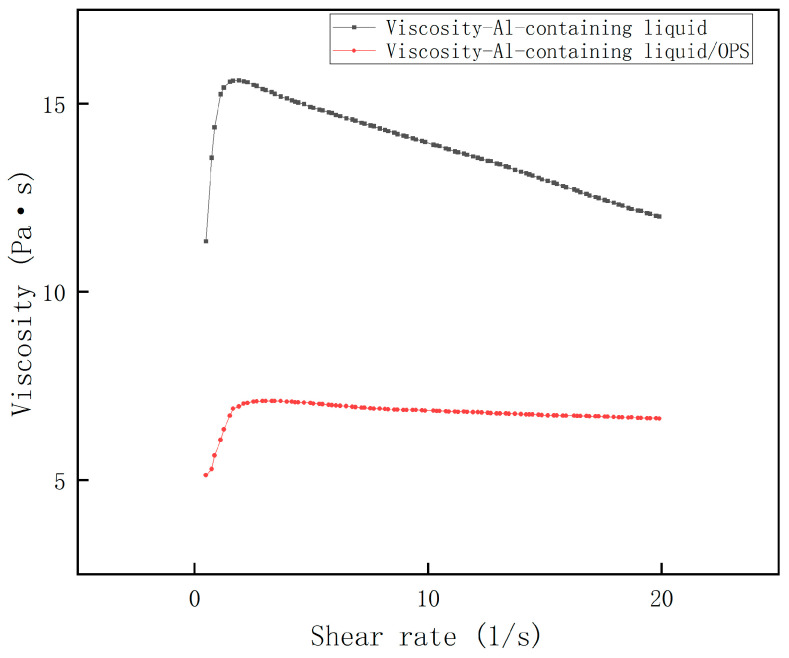
Viscosity curve of Al + HTPB/DOS system.

**Figure 4:**
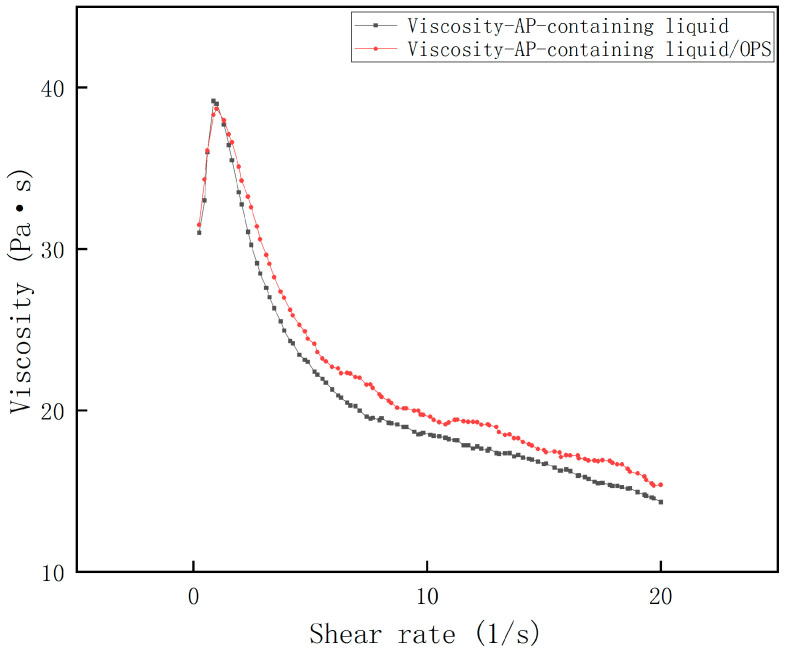
Viscosity curve of AP + HTPB/DOS system.

The shear rate–viscosity curves of Al-containing and AP-containing fluids were measured, and the results are shown in Figure 3 and Figure 4. It can be seen that all the rheological profiles of the slurries satisfy the rheological characteristics of the polymer fluids mentioned above, and all of them reach the maximum viscosity at a shear rate close to 1 s^−1^, followed by a significant decrease. Obviously, the viscosity of the Al-containing fluid decreased dramatically after the addition of OPS, and the decrease was often more than 50%; while the viscosity of the AP-containing fluid did not change much after the addition of OPS, and even showed an increase of about 7% in the range of the curve after crossing the peak value. From this, it can be initially judged that the viscosity reduction effect of OPS on the blank formulation slurry is mainly accomplished by regulating the interfacial properties of Al and the liquid system.

**Figure 5:**
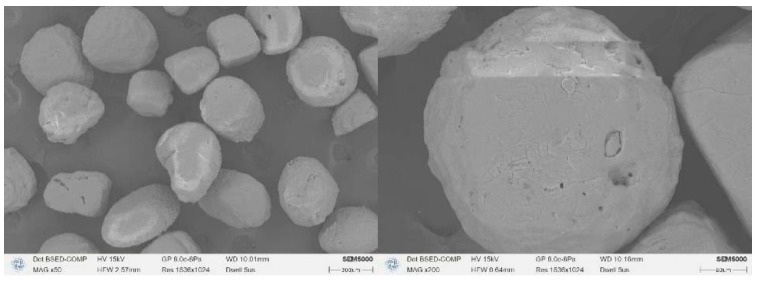
SEM images of AP particles.

**Figure 6:**
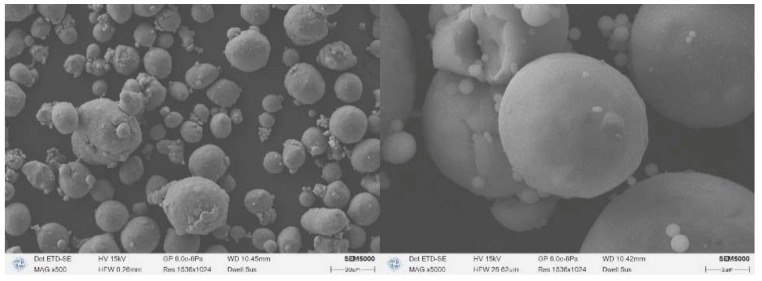
SEM images of Al particles.

AP forms white crystals with a density of 1.94 g/cm^3^; Al forms gray crystals with a density of 2.70 g/cm^3^. The surface morphology of class III AP and 29 μm Al used in this study is shown in Figure 5 and Figure 6, and it is obvious that Al has a higher degree of sphericity. It is generally accepted that the sphericity of micron-sized aluminum powders can be up to 0.98 or more, which can be approximated as spherical, while the AP sphericity is relatively low [23,24,25]. The Al powder and AP sphericity chosen for the experiment were 0.95 and 0.53, respectively. The different surface morphologies of the two solid-phase particles may lead to different coating effects of the binder system on the particles. At the microscopic level, the dispersibility of the 29 μm aluminum powder selected for the experiment was slightly poor, with a small range of local agglomerations, and the effect of viscosity reduction was more obvious after surfactant dispersion. Strong surface adsorption and self-agglomeration existed between the experimentally selected 29 μm Al particles, and the internal friction between the particles after mixing with HTPB to form a suspension was also more obvious [26]. Under the premise that the particle size of class III AP is larger than 29 μm Al, the viscosity of the AP-containing liquid is still significantly higher than that of the Al-containing liquid, which is attributed to the high sphericity of Al, which can form a better coating with HTPB/DOS in the case of a blank formulation.

**Figure 7:**
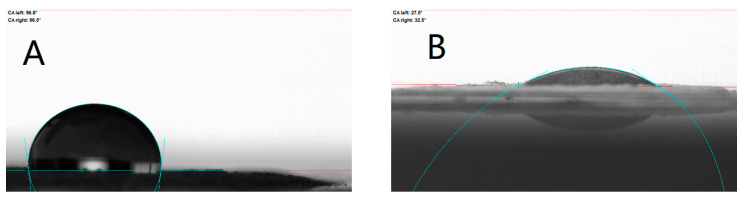
Contact angle of slurry in Al. (**A**) Al-containing liquid; (**B**) Al-containing liquid/OPS.

**Figure 8:**
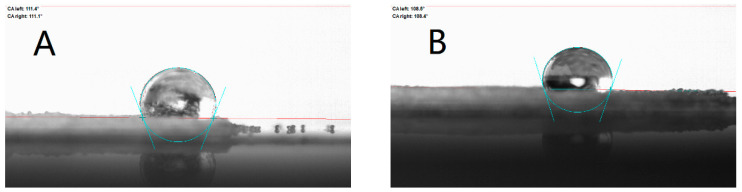
Contact angle of slurry in AP. (**A**) AP-containing liquid; (**B**) AP-containing liquid/OPS.

Furthermore, contact angle tests were performed for various interfaces in the slurry system, and the results are shown in Figure 7 and Figure 8. A smaller contact angle means better wettability at the interface between the solid phase and the liquid droplet. The surface interface is a high-energy region of the object, and its presence is related to surface tension. Surface tension is the tension formed at the surface of a liquid when the cohesive force of the molecules on the surface is greater than the intermolecular gravitational force. Larger surface tension is not favorable for wetting, which is manifested in the slurry by the inability of the solid phase particles to be uniformly dispersed and to form an effective envelope with the liquid, thereby increasing the internal resistance and impeding the flow of the slurry, thereby increasing the viscosity [27]. Therefore, the contact angle is also a measure and an important indicator of rheology, especially rheological properties associated with solid–liquid interfaces. When the contact angle is greater than 90°, the droplets are not wetted to the contact surface, at which point the surface tension is higher and the rheological properties can be severely limited. The contact angle test showed that the addition of OPS decreased the contact angle of the slurry on the Al surface from about 97° to about 30°, and the interface was changed from unwetted to wetted; while the addition of OPS to the slurry had little effect on the contact angle of AP, given that OPS has strong surface activity, the interfacial contact angle between AP and the slurry was reduced only by about 3°, and AP and the slurry were still unwetted after the addition of OPS, which indicated that the addition of OPS did not significantly improve the interfacial properties between AP and the liquid phase. Accordingly, it can be judged that OPS improves the rheological properties of the slurry by improving the interfacial wetting between the liquid system and Al. The results of the contact angle tests are shown in Table 2.

The active ingredients in OPS additives are polyalkenyl polyamines. Polyalkenyl polyamines in their molecular chains often exist at the same time amine groups, polyoxyethylene, and other functional groups, with unsaturated bonds and amine groups, coupled with the arrangement of the functional groups to form a multi-arm structure so that polyalkenyl polyamines tend to have surface activity [28]. Specific advantages of polyene polyamines as surface-active substances include the following. (1) Polar groups: the amine groups in polyalkenyl polyamines have strong polarity and easily form hydrogen bonds, thus regulating the inter-interfacial interactions. (2) Self-assembly properties: self-assembly properties are an important requirement for highly efficient surfactants at present, and polyalkenyl polyamines can form an orderly arranged structure at the interfacial position through self-assembly, thus forming an effective coating for the particles and preventing the interface from unnecessary rupture, thus showing strong surface activity.

## 4. Conclusions

The process aids produced a positive and significant improvement in the rheological properties of the three-component HTPB propellant slurries, which facilitated the development of high-solid-content composite propellant formulations, and will ultimately significantly improve the propellant energy performance. The conclusions are as follows:

- Surfactant OPS showed significant improvement in the rheological properties of the three-component HTPB slurry, and the addition of the 0.1% mass fraction OPS to the liquid system of a blank formulation with 84% solids resulted in an average reduction in system viscosity. The average viscosity of the slurry dropped from 74.49 Pa·s to 68.83 Pa·s.
- Surfactant OPS affects the rheological properties by improving the properties of the Al-HTPB–DOS interface through improving the wetting of the Al-HTPB/DOS interface and promoting the encapsulation of particles by the liquid to reduce the viscosity. The contact angle decreased from 96.65° to 30.05° with the addition of OPS to the slurry.

## Figures and Tables

**Table 1:** Slurry composition comparison.

Abbreviations	Component (Mass Ratio)
Full-component formulations	HTPB/DOS/Class III AP/Al/Fine AP (3:1:3.38:1.19:0.43)
Full-component formulations/OPS	HTPB/DOS/Class III AP/Al/Fine AP/OPS (3:1:3.38:1.19:0.43:0.09)
Al-containing liquid	HTPB/DOS/Al (3:1:5)
AP-containing liquid	HTPB/DOS/Class III AP (3:1:5)
Al-containing liquid/OP	HTPB/DOS/Al/OPS (3:1:5:0.009)
AP-containing liquid/OPS	HTPB/DOS/Class III AP/OPS (3:1:5:0.009)

**Table 2:** Data of contract angle.

Slurry	Left of the Drop	Right of the Drop	Average
Al-containing liquid	96.80°	96.50°	96.65°
Al-containing liquid/OPS	27.50°	32.60°	30.05°
AP-containing liquid	111.40°	111.10°	111.25°
AP-containing liquid/OPS	108.50°	108.40°	108.45°

## Data Availability

The original contributions presented in this study are included in the article. Further inquiries can be directed to the corresponding author(s).
